# *Lycopus maackianus* Makino MeOH Extract Exhibits Antioxidant and Anti-Neuroinflammatory Effects in Neuronal Cells and Zebrafish Model

**DOI:** 10.3390/antiox11040690

**Published:** 2022-03-31

**Authors:** Hwan Lee, Zhiming Liu, Linsha Dong, Sun Hee Cheong, Dong-Sung Lee

**Affiliations:** 1 College of Pharmacy, Chosun University, Dong-gu, Gwangju 61452, Korea; ghksdldi123@hanmail.net (H.L.); lzmqust@chosun.kr (Z.L.); donglinsha011@chosun.kr (L.D.); 2 Department of Marine Bio-Food Sciences, Chonnam National University, Yeosu 59626, Korea; sunny3843@jnu.ac.kr

**Keywords:** *Lycopus maackianus*, antioxidant activity, anti-neuroinflammatory effects, BV2 microglia, HT22 hippocampus, zebrafish

## Abstract

*Lycopus maackianus* Makino belongs to the Labiatae family and is used in traditional medicine to manage postpartum edema and boils. However, few studies on its antioxidant and anti-inflammatory effects have been conducted. Here, the compounds in *L. maackianus* methanol (MeOH) extract were profiled using ultra-high-performance liquid chromatography–time-of-flight high-resolution mass spectrometry analysis. The antioxidant activity of *L. maackianus* MeOH extract was shown to increase in a concentration-dependent manner by investigating the 2,2-diphenyl-1-picrylhydrazyl and 2,2′-azino-bis(3-ethylbenzothiazoline-6-sulfonic acid) radical scavenging activity. Next, in lipopolysaccharide-treated BV2 cells, *L. maackianus* extract inactivated the nuclear factor-kappa B pathway, inhibiting nitric oxide, prostaglandin E2, interleukin-6, and tumor necrosis factor-α production and inducible nitric oxide synthase and cyclooxygenase-2 protein expression. Furthermore, *L. maackianus* extract protected against oxidative stress-induced cellular damage in glutamate-stimulated HT22 cells. *L. maackianus* MeOH extract induced heme oxygenase-1 expression and increased the translocation of nuclear factor E2-related factor 2 in the nucleus, thus exhibiting antioxidant and anti-inflammatory effects. Moreover, the in vivo antioxidant and anti-inflammatory effects of the extract were demonstrated in a zebrafish (*Danio rerio*) model treated with hydrogen peroxide and lipopolysaccharide. MeOH *L. maackianus* extract showed antioxidant and anti-neuroinflammatory effects by increasing the expression of heme oxygenase-1, establishing its therapeutic potential for neuroinflammatory diseases.

## 1. Introduction

The relationship between oxidative damage and inflammation is implicated in the pathogenesis of neurodegenerative diseases [1]. When an oxidative imbalance occurs due to an abnormality in the body, the antioxidant system is dysregulated, leading to oxidative stress and the production of reactive oxygen species (ROS) and reactive nitrogen species (RNS) (nitric oxide (NO), hydrogen peroxide (H_2_O_2_), superoxide ion (O_2_^•−^)) [2]. The accumulation of ROS and RNS leads to apoptosis, resulting in protein and organelle damage; mitochondrial membrane disruption; and neuronal cell death [3,4].

The pathogenesis of neurodegenerative diseases that start with inflammation is similar to that of oxidative stress. Microglia and macrophages in the brain are immune cells that play important roles in mediating inflammatory responses in the central nervous system (CNS) [5]. Lipopolysaccharide (LPS)-stimulated microglia express pro-inflammatory cytokines (tumor necrosis factor (TNF)-α and interleukin (IL)-6), pro-inflammatory mediators (prostaglandin E2 (PGE_2_) and NO), and pro-inflammatory proteins (inducible nitric oxide synthase (iNOS) and cyclooxygenase (COX)-2), leading to inflammation-related neuronal dysfunction [6]. The inhibitor of the kappa B (IκB) protein binds to the cytoplasmic nuclear factor-kappa B (NF-κB) heterodimer under normal conditions; however, overactivation induces IκB degradation via the ubiquitin–proteasome system, resulting in the translocation of the free NF-κB heterodimer to the nucleus. This induces the expression of inflammatory mediators and cytokines and reveals that the regulation of NF-κB translocation is recognized as a strategy for suppressing microglial inflammation through overactivation [7,8,9].

Nuclear factor E2-related factor 2 (Nrf2) is a known regulator of the antioxidant response and an upstream transcription factor that induces the expression of antioxidant enzymes [10]. Among the antioxidant enzymes expressed in association with Nrf2, heme oxygenase (HO)-1 is known to exhibit strong antioxidant and anti-neuroinflammatory effects by catalyzing the degradation of carbon monoxide (CO), iron, and biliverdin/bilirubin [11,12]. Several studies have reported that the activation of Nrf2-antioxidant signaling attenuates NF-κB-mediated inflammatory responses and that the upregulation of the Nrf2/HO-1 axis prevents neuronal cell death in activated microglia [13,14]. This allows the identification of the NF-κB and HO-1 pathways that target the anti-inflammatory effects of antioxidant enzymes.

*Lycopus maackianus* is a perennial herb found in East Asia that belongs to the Labiatae family. It grows up to 0.6 m in height, flowers bloom in July–September, and seeds ripen in August–November. Growing in wetlands, the species is hermaphrodite (has both male and female organs). Dried *L. maackianus* has been used in oriental medicine to treat post-partum edema, boils, and snake venom poisoning. In addition, the young leaves are used for food. Lavender, used for aromatherapy, and lemon balm, used for wound healing, are famous medicinal plants belonging to the Labiatae family [15,16,17,18]. In addition, *Lycopus lucidus* Turcz, belonging to the same family as *L. maackianus*, is an excellent herbal medicine known as “Zelan” in China. *L. lucidus* has long been used to promote blood circulation, manage hematomas, and treat inflammatory diseases [19]. In addition, various active studies on fatty liver, neuroinflammation, and dermatitis have been reported using *L. lucidus* [20,21,22]. Likewise, *L. maackianus* is a medicinal plant that has long been used in China for its healing properties [19]. However, there have been few reports on *L. maackianus*.

The purpose of this study was to analyze the components of the methanol (MeOH) extract of *L. maackianus*, which are unknown, and to reveal the extract’s therapeutic potential against oxidative damage and inflammation. To this end, the anti-neuroinflammatory action of *L. maackianus* MeOH extract on LPS-stimulated BV2 microglia was investigated. In addition, the neuroprotective effects of this extract on glutamate-induced oxidative stress in HT22 hippocampal cells were investigated, and the antioxidant and anti-neuroinflammatory effects of *L. maackianus* MeOH extract were also investigated by stimulating zebrafish with H_2_O_2_ and LPS.

## 2. Materials and Methods

### 2.1. Preparation of Extract from L. maackianus

*L. maackianus* samples (voucher specimen; CU1049-17) collected from the Herb Garden of Chosun University, Republic of Korea, were authenticated in January 2015 by Professor Eun-ran Woo of the Chosun University. As for the extraction method, dry *L. maackianus* (100 g) was extracted with MeOH at 80 °C under reflux for 3 h. The ratio of raw material to extraction solvent was maintained at 1 g/30 mL; it was extracted twice and evaporated to obtain 15 g of Makino MeOH extract.

### 2.2. Materials

Cell culture reagents, including fetal bovine serum (FBS), Dulbecco’s modified Eagle medium (DMEM), and Roswell Park Memorial Institute (RPMI)-1640 medium, were purchased from Gibco BRL Co. (Grand Island, NY, USA). Primary antibodies against p65, COX-2, iNOS, Nrf2, HO-1, proliferating cell nuclear antigen (PCNA), and β-actin were purchased from Cell Signaling Technology (Danvers, MA, USA). All secondary antibodies were purchased from Millipore (Billerica, MA, USA). All of the enzyme-linked immunosorbent assay (ELISA) kits were purchased from R&D Systems (Minneapolis, MN, USA). All other chemicals were purchased from Sigma-Aldrich (St. Louis, MO, USA).

### 2.3. Metabolite Profiling Analysis

Component profiling was performed using ultra-high-performance liquid chromatography (UHPLC) under the following conditions. The analytical equipment used was a Dionex Ultimate 3000 (Thermo Dionex, Sunnyvale, CA, USA) equipped with a Waters CORTECS T3 column (1.6 μm, 2.1 × 150 mm; Waters Technologies, Milford, MA, USA) maintained at 45° for analysis. During chromatography, mobile phase solvent A was 0.1% formic acid in water (*v*/*v*) and solvent B was 0.1% formic acid in acetonitrile (*v*/*v*), which was run in a gradient system as follows: 0 min (1% B), 0–10 min (5% B), 10–30 min (30% B), 30–50 min (100% B), 50–54 min (100% B), and 55–60 min (1% B). For the sample used for analysis, 3 μL of *L. maackianus* MeOH extract prepared at a concentration of 20 mg/mL using 80% MeOH was injected. The flow rate was set at 0.3 mL/min. High-resolution mass spectrometry (HRMS) was performed on a Triple time-of-flight (TOF) 5600+ (AB Sciex, Framingham, MA, USA) using an electrospray source in the positive and negative ion modes (electrospray ionization (+)−MS and electrospray ionization (−)−MS). The MS instrument settings used were as follows: source temperature, 500 °C; MS scan range, 100 to 2000 *m*/*z* (MS); 30 to 2000 *m*/*z* (MS/MS); gas pressure, 50 psi (nebulizing, heating) and 25 psi (curtain); ion spray voltage, 5500 V (positive) and 4500 V (negative); and collision energy, 35 ± 15 eV. Data collection and processing were performed using Elements Viewer version 2.1 and MS was performed in both positive and negative ion modes.

### 2.4. 2,2-Diphenyl-1-picrylhydrazyl (DPPH) Radical Scavenging Activity

For DPPH analysis, 0.20 mM was prepared by dissolving the DPPH solution in MeOH. In addition, extracts at three concentrations (0.5, 1, and 2 mg/mL) were prepared. The prepared DPPH solution and each extract were mixed in a ratio of 3:1. After reacting the mixed sample in the dark for 30 min, absorbance was measured at 517 nm. The DPPH radical scavenging activity was expressed as an inhibition rate (%) by substituting it in the following formula:

DPPH radical scavenging activity (%) = (A_control_ − A_sample_)/A_control_ × 100 (1)

where A_sample_ and A_control_ is the absorbance of the test sample and control, respectively.

### 2.5. 2,2′-Azino-bis(3-ethylbenzothiazoline-6-sulfonic Acid) (ABTS) Radical Scavenging Activity

For ABTS assay, the reagents were prepared by mixing ABTS (7 mM) and potassium persulfate (140 mM). In order to induce the formation of free radicals, the mixed reagents were reacted in the dark for 16 h and then diluted with water. The diluted reagent and extract were mixed in a 1:1 ratio and dispensed on a microplate. After reacting at room temperature for 6 min, absorbance was measured at 734 nm. The control group used 100% MeOH. The ABTS radical scavenging activity was expressed as an inhibition rate (%) by substituting it in the following formula:

ABTS radical scavenging activity (%) = (Blank O.D. − Sample O.D.)/Blank O.D. × 100.(2)

### 2.6. Cell Culture and Viability Assay

BV2 cells (5 × 10^5^ cells/mL) were cultured in RPMI-1640 containing 10% heat-inactivated FBS and antibiotic–antimycotic solution (Thermo Fisher Scientific, Waltham, MA, USA). HT22 hippocampus were cultured in DMEM containing 10% heat inactivated FBS and antibiotic–antifungal solution. The cell culture conditions were incubated at 37 °C and 5% CO_2_ for 12–24 h. Mitochondrial reductase reduces the tetrazolium salt 3-[4,5-dimethylthiazol-2-yl]-2,5-diphenyltetrazolium bromide (MTT) to formazan crystals. Using this, the effect of *L. maackianus* extract on cell viability was measured. To measure cell viability, 5 mg/mL MTT was treated with each cell suspension (1 × 10^5^ cells/mL) to form formazan for 4 h. The formed formazan was dissolved in DMSO and absorbance was measured at 540 nm.

### 2.7. Determination of Nitrite Levels

The nitrite levels were evaluated as an indicator of NO secretion in BV2 cells. The nitrite levels were measured at 570 nm after the cell culture solution and Griess reagent were mixed at the same volume and allowed to react.

### 2.8. Determination of PGE_2_ Levels

Briefly, the BV2 cells were cultured in 48-well plates (1 × 10^5^ cells/mL) and pre-treated with different concentrations of *L. maackianus* extract for 3 h. Subsequently, the BV2 cells were induced with LPS (0.5 μg/mL for 24 h). To remove particulate matter, supernatants were collected. Then, the PGE_2_ levels were measured using a specific ELISA kit following a previously described method [23].

### 2.9. Determination of IL-6 and TNF-α Levels

Briefly, the BV2 cells were seeded in 24-well culture plates (5 × 10^5^ cells/well) and pre-treated with different concentrations of *L. maackianus* extract for 3 h. Next, the cells were stimulated with LPS (0.5 μg/mL) for 24 h. After incubation, the levels of IL-6 and TNF-α were measured in the supernatant collected from the medium using cytokine ELISA kits according to the manufacturer’s instructions.

### 2.10. Western Blot Analysis

The protein levels were determined using Western blot analysis. To this end, the cells were lysed, and the protein concentration was measured using protein assay dye reagent concentrate (#5000006; Bio-Rad Laboratories, Hercules, CA, USA). Western blotting was performed as previously described [23].

### 2.11. Preparation of Cytosolic and Nuclear Fractions

A nuclear extraction kit (Cayman, Ann Arbor, MI, USA) was used to separate cytosolic and nuclear fractions. Each extracted fraction was lysed according to the protocol provided by the manufacturer.

### 2.12. NF-κB Localization and Immunofluorescence

To detect the localization of NF-κB, the following experiments were performed. BV2 cells were grown on Lab-Tek II chamber slides and treated with *L. maackianus* extract for 3 h, and then treated with LPS for 0.5 h. The staining process was performed as previously described [24]. After staining, the cells were visualized and photographed using a Zeiss fluorescence microscope (Provis AX70; Olympus Optical Co., Tokyo, Japan).

### 2.13. Origin and Maintenance of Parent Zebrafish

Adult zebrafish were purchased from a commercial dealer (Seoul Aquarium, Seoul, Korea) and stored in a 3-L acrylic water tank at 28.5 °C with a 14 h/10 h light–dark cycle. For the zebrafish culture, a tetramine flake diet supplemented with live brine shrimp (*Artemia salina*; Sewhapet Food Co., Seoul, Korea) was supplied three times a day, 6 d a week. Embryos were obtained through spontaneous spawning induced by light in the morning. Embryo collection was completed within 30 min. The zebrafish were treated in accordance with the Chonnam National University Guidelines for the Care and Use of Laboratory Animals. The experimental protocols used in this study were approved by the Animal Ethics Committee (No. CNU IACUC-YS-2020-8) of Chonnam National University.

### 2.14. Determination of ROS Levels in HT22 Cells

HT22 cells were cultured in 48-well plates (1 × 10^5^ cells/mL), and pre-treated with different concentrations of *L. maackianus* extract for 3 h. HT22 cells were induced with glutamate (10 mM for 24 h), then the media was removed and the cells were loaded with 10 μM DCFH-DA in phosphate buffered saline (PBS). The plate was returned to a 37 °C incubator and incubated for 20 min. After washing with PBS, the fluorescence intensity was measured at an excitation wavelength of 495 nm and an emission wavelength of 529 nm in a multi-mode reader (BiotekTM Synergy H1 Hybrid Multi-Mode Reader, Winooski, VT, USA).

### 2.15. LPS and H_2_O_2_ Treatment of Zebrafish Embryos

Synchronized zebrafish embryos were collected and arranged in groups of six embryos in 12-well plates containing 2 mL of embryo medium for 7–9 h post fertilization. Then, the extracts were treated by groups and incubated. Next, to induce oxidative stress and inflammation, 5 mM H_2_O_2_ and 10 μg/mL LPS (final concentration) were treated, incubated for 1 h, and then transferred to fresh embryo medium maintained at 28.5 °C. All experiments were measured in zebrafish 7 days after embryo fertilization.

### 2.16. Cell Death Measurement and Image Analysis in Zebrafish Embryos

The zebrafish embryos were transferred to a 96-well plate at 7 dpf and treated with acridine orange solution (7 g/mL). After treatment, they were incubated for 30 min in the dark at 28.5 °C. The zebrafish embryos were then rinsed in fresh embryo medium and anesthetized using 2-phenoxy ethanol (1/500 dilution) before observation, and photographed using a CoolSNAP-Pro color digital camera (Olympus, Tokyo, Japan). The fluorescence intensity of individual larvae was quantified using the ImageJ software.

### 2.17. Determination of ROS Levels and Image Analysis in Zebrafish Embryos

The ROS production in the zebrafish was analyzed using dichloro-dihydro-fluorescein diacetate (DCFH-DA), known as a fluorescent probe dye. On day 7, the zebrafish embryos were moved to a well plate and treated with DCFH-DA (20 μg/mL). They were then incubated for 1 h in a dark room at 28.5 °C. The zebrafish embryos were then washed in fresh medium and anesthetized using tricaine methane sulfonate prior to observation. Photos were taken using a Moticam color digital camera (Motix, Xiamen, China). Fluorescence intensity was quantified using a LS-5B spectrofluorometer (PerkinElmer, Norwalk, CT, USA).

### 2.18. Determination of NO Levels and Image Analysis in Zebrafish Embryos

The NO production in the zebrafish model was analyzed using the diaminofluorophore 4-amino-5-methylamino-2′,7′-difluorofluorescein diacetate (DAF-FM-DA). On day 7, the zebrafish embryos were moved to well plates and treated with DAF-FM-DA (5 μM). They were then incubated for 1 h in a dark room at 28.5 °C. The next experimental process was carried out using the same methods as described in Section 2.17: Determination of ROS Levels and Image Analysis in Zebrafish Embryos.

### 2.19. Statistical Analyses

A minimum of three independent experiments were conducted to achieve the results described herein. Data are expressed as the mean ± SD deviation of three independent experiments. All data were evaluated using one-way analysis of variance followed by the Tukey’s multiple comparison test. Statistical analyses were performed using GraphPad Prism version 5.01 (GraphPad Software, Inc., San Diego, CA, USA).

## 3. Results

### 3.1. UHPLC–TOF-HRMS Analysis of L. maackianus MeOH Extract

Ten compounds were profiled using UHPLC–TOF-HRMS chromatogram analysis of the MeOH extract of *L. maackianus*. The main peaks attributed to the chromatogram were observed in both the positive and negative modes using electrospray ionization–MS. Moreover, to identify the compound, the ion peak was compared with those found in literature, along with the MS information of the compound. The UHPLC–TOF-HRMS analysis revealed that the 10 compounds consisted of phenols, saponins, glycosides, alkaloids, flavonoids, and terpenoids (Table 1).

### 3.2. Effect of L. maackianus Extract on DPPH and ABTS Radical Scavenging Activity

**Figure 1:**
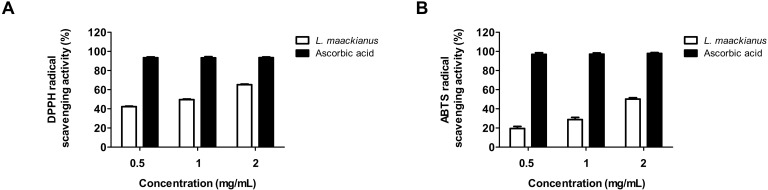
Effect of *Lycopus maackianus* MeOH extract on 2,2-diphenyl-1-picrylhydrazyl (DPPH) (**A**) and 2,2′-azino-bis(3-ethylbenzothiazoline-6-sulfonic acid) (ABTS) (**B**) radical scavenging activity. Ascorbic acid was used as the positive control. Data are presented as the mean ± SD of three independent experiments.

To investigate the antioxidant activity of the MeOH extract of *L. maackianus*, the free radical scavenging activities of DPPH and ABTS were measured. A DPPH radical scavenging assay is utilized to measure the reducing power because of the electron-donating ability of DPPH, which is a relatively stable radical, and is a widely used method to test antioxidant activity. Ascorbic acid, an antioxidant, was used as a positive control. Using this method, it was shown that the free radical scavenging activity of *L. maackianus* MeOH extract increased in a concentration-dependent manner (Figure 1A). The next objective was to analyze the antioxidant ability of the MeOH extract of *L. maackianus* using ABTS assay, which is another method for measuring antioxidant activity. The results showed that ABTS radical scavenging activity also increased in a concentration-dependent manner (Figure 1B).

### 3.3. Effects of L. maackianus Extract on Levels of Pro-Inflammatory Mediators and Cytokines in LPS-Induced BV2 Cells

**Figure 2:**
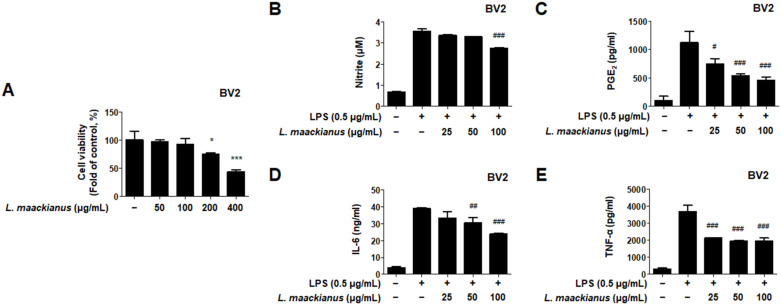
Effect of the MeOH extract of *Lycopus maackianus* on cytotoxicity (**A**), nitric oxide (NO) (**B**), prostaglandin E2 (PGE_2_) (**C**), interleukin (IL)-6 (**D**), and tumor necrosis factor (TNF)-α (**E**) levels in BV2 cells. Cells were pretreated with the indicated concentrations of *L. maackianus* extract for 3 h and stimulated for 24 h in the presence or absence of lipopolysaccharide (LPS; 0.5 μg/mL). Bars represent the mean ± standard deviation of three independent experiments. Untreated control groups were included. * *p* < 0.05, *** *p* < 0.001 compared with control group. ^#^ *p* < 0.05, ^##^ *p* < 0.01, ^###^ *p* < 0.001 compared with LPS-treated group.

The anti-neuroinflammatory efficacy of LPS-induced BV2 microglia was investigated by treating them with the MeOH extract of *L. maackianus*. First, a toxicity evaluation was performed by applying the *L. maackianus* extract at a concentration of 50–400 μg/mL to determine the treatment concentration (Figure 2A). It was shown that toxicity occurred at 200 μg/mL. Thus, the maximum treatment concentration of the extract was set at 100 μg/mL to determine its inhibitory effect on nitrite production. As a positive control, sulfuretin, which is known to have an excellent anti-inflammatory effect, was used at a concentration of 20 μM for comparison. The effect of the MeOH extract of *L. maackianus* was superior to that of the control at 100 μg/mL (Figure 2B). Subsequently, we demonstrated that the production of PGE_2_ was inhibited in a concentration-dependent manner (Figure 2C). In addition, *L. maackianus* extract inhibited the production of IL-6 and TNF-α in a concentration-dependent manner (Figure 2D,E). These results show that the MeOH extract of *L. maackianus* significantly inhibited the production of inflammatory substances in LPS-induced BV2 microglia.

**Figure 3:**
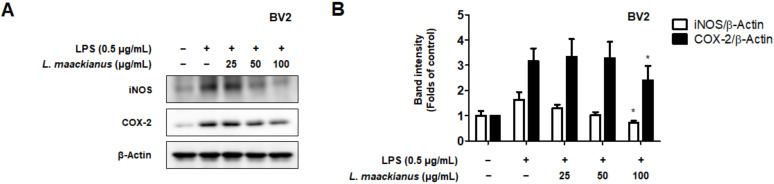
Effect of *Lycopus maackianus* methanol (MeOH) extract on inducible nitric oxide synthase (iNOS) and cyclooxygenase (COX)-2 protein levels in BV2 cells (**A**). Western blotting was performed after treating the cells with *L. maackianus* MeOH extract at the indicated concentrations for 3 h, followed by stimulation with lipopolysaccharide (LPS; 0.5 µg/mL) for 24 h. The immunoblot was quantified using the ImageJ software (**B**). Band intensities were normalized to those of β-actin. Data are the average of three independent experiments. Untreated control groups were included. * *p* < 0.05 compared to the LPS treatment group.

Moreover, we investigated whether these inhibitory effects on inflammatory mediators were involved in the regulation of iNOS and COX-2 expression. LPS treatment increased iNOS and COX-2 levels; however, *L. maackianus* extract inhibited the LPS-induced increase in iNOS and COX-2 levels in a concentration-dependent manner (Figure 3).

### 3.4. Effects of L. maackianus Extract on NF-κB Activation in LPS-Induced BV2 Cells

**Figure 4:**
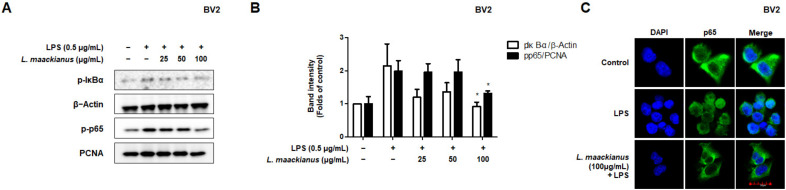
Effects of *Lycopus maackianus* extract on nuclear factor-kappa B (NF-κB) p65 activation in BV2 cells. (**A**) Western blotting was performed after treating the cells with *L. maackianus* MeOH extract at the indicated concentrations for 3 h, followed by stimulation with lipopolysaccharide (LPS; 0.5 µg/mL) for 0.5 h. (**B**) The immunoblot was quantified using the ImageJ software. Band intensities were normalized to those of β-actin. (**C**) NF-κB (p65) localization in BV2 cells were visualized and photographed using a Zeiss fluorescence microscope. Data are the average of three independent experiments. Untreated control groups were included. * *p* < 0.05 compared with LPS-treated group.

During the inflammatory response, abnormal NF-κB signaling leads to the excessive secretion of pro-inflammatory mediators and cytokines. The inhibitory effect on NF-κB activation in LPS-stimulated BV2 cells was investigated using Western blotting. To investigate this inhibitory effect, the cytosolic and nuclear fractions were extracted from LPS-induced BV2 cells treated with *L. maackianus* extract at various concentrations (25–100 µg/mL). As shown in Figure 4A, the *L. maackianus* extract inhibited the degradation of the nuclear factor of kappa light polypeptide gene enhancer in B-cells inhibitor, alpha (IκBα), and the nuclear translocation of p65 in a concentration-dependent manner (Figure 4A). In addition, LPS increased the DNA-binding activity of NF-κB in the extract (Figure 4B). Immunofluorescence analysis also showed that LPS induced NF-κB (p65) translocation into the nucleus, and *L. maackianus* extract significantly inhibited the translocation of NF-κB compared to that seen in the controls (Figure 4C). These results suggest that *L. maackianus* extract inhibits the nuclear translocation of NF-κB (p65) induced by LPS stimulation by preventing IκBα phosphorylation.

### 3.5. Effects of L. maackianus Extract on Glutamate-Induced Oxidative Stress in HT22 Cells

**Figure 5:**
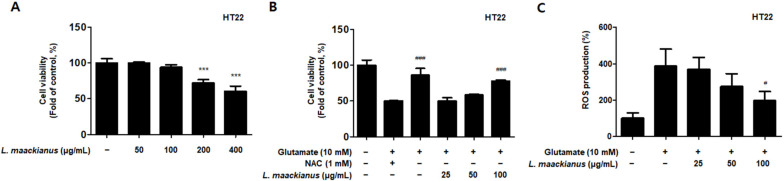
Neuroprotective effect of the MeOH extract of *Lycopus maackianus* on glutamate-induced HT22 hippocampal cells. Cells were pretreated with the indicated concentrations of extract for 3 h and analyzed in the absence (**A**) or presence (**B**) of glutamate (10 mM) for 24 h. Reactive oxygen species (ROS) generation inhibitory effect (**C**) of *L. maackianus* MeOH ex-tract in a glutamate-treated HT22 cell. Data are the average of three independent experiments. Untreated control groups were included. *** *p* < 0.001 compared with control group. ^#^ *p* < 0.05, ^###^ *p* < 0.001 compared with glutamate- and *L. maackianus* MeOH-treated group.

Glutamate is an important neurotransmitter in the CNS. However, when glutamate is over-secreted because of various causes, it triggers oxidative stress, which leads to neuronal cell death. HT22 hippocampal cells are responsible for memory, and when treated with high concentrations of glutamate, apoptosis occurs because of oxidative stress. Here, it was further investigated whether the MeOH extract of *L. maackianus* exhibits neuroprotective effects against oxidative stress-induced cytotoxicity in glutamate-induced HT22 hippocampal cells. First, to determine the sample treatment concentration, the cytotoxicity was evaluated for each concentration (50–400 µg/mL; Figure 5A). Toxicity occurred at 200 µg/mL, and thus, the neuroprotective effect on oxidative stress was investigated by setting the highest concentration at 100 µg/mL. The MeOH extract of *L. maackianus* showed concentration-dependent neuroprotective effects in glutamate-induced HT22 hippocampal cells (Figure 5B). Meanwhile, Figure 5C shows the results of the ROS production evaluation. Compared to that in the control group, ROS production was significantly increased in the glutamate treatment group. However, ROS production in the extract group was significantly reduced.

### 3.6. Effects of L. maackianus Extract on HO-1 Expression and Nrf2 Nuclear Translocation

**Figure 6:**
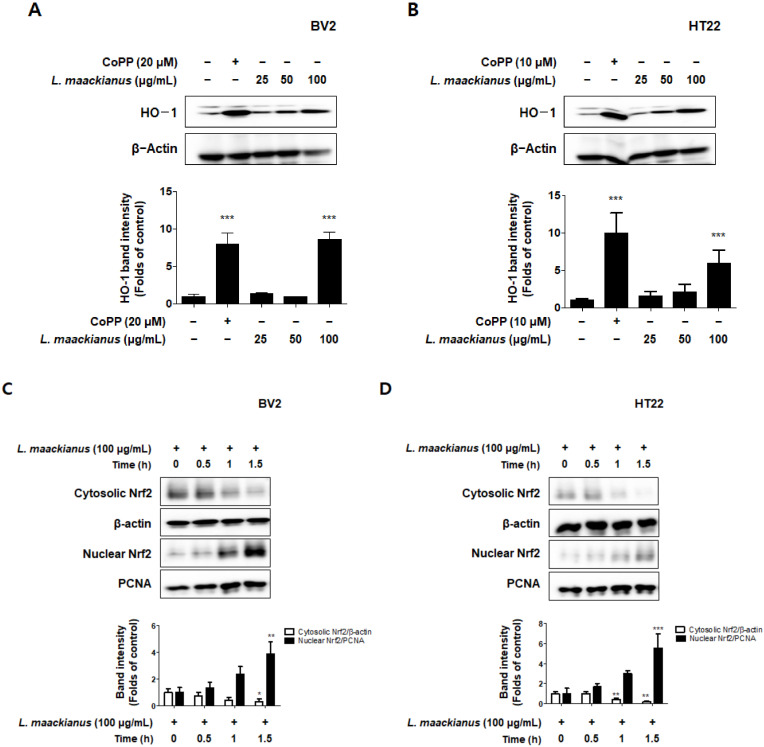

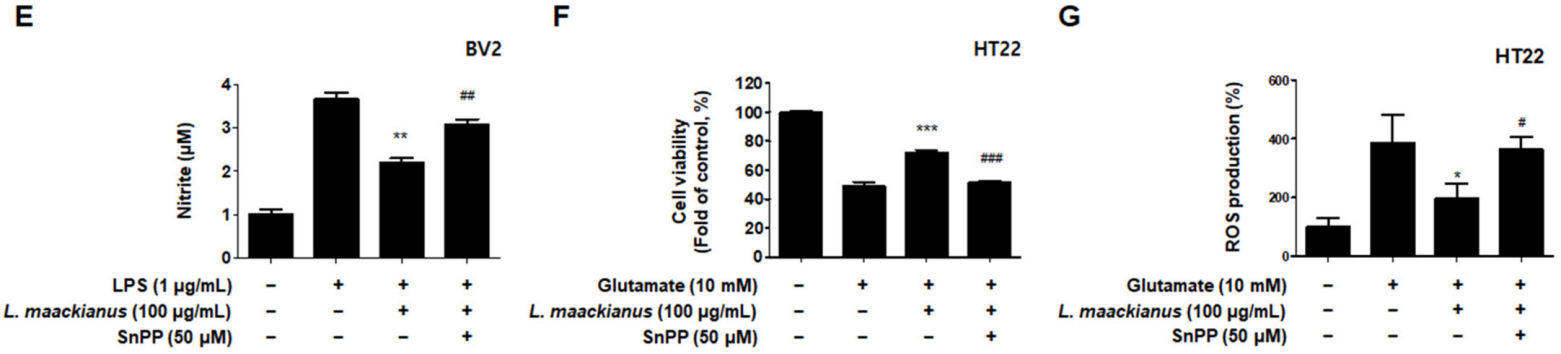
Effect of *L. maackianus* extract on nuclear factor E2-related factor 2 (Nrf2)/heme oxygenase (HO)-1 protein expression in BV2 and HT22 cells. HO-1 expression levels (**A**,**B**) were determined using Western blotting after cells were incubated for 12 h in the presence or absence of cobalt protoporphyrin (CoPP) at the concentrations indicated for each sample. To measure Nrf2 expression (**C**,**D**), cells were treated with the extract for 0.5, 1, and 1.5 h, and the cytoplasm and nucleus were fractionated using the Cayman nuclear extraction kit. Thereafter, protein expression was determined using Western blotting. Band intensities were normalized to those of β-actin or proliferating cell nuclear antigen (PCNA). In addition, the effect of Nrf2/HO-1 expression on nitrite inhibition (**E**), neuroprotective effects (**F**), and ROS production (**G**) was investigated. Cells were pretreated with the extract alone or with SnPP (50 μM) for 3 h and then stimulated with LPS (1 μg/mL) and glutamate (10 mM) for 24 h. Subsequently, nitrite levels, cell viability and ROS were measured. Data are presented as the mean ± standard deviation for three independent experiments. Unprocessed controls were included. * *p* < 0.05, ** *p* < 0.01, *** *p* < 0.001 compared to control. ^#^ *p* < 0.05, ^##^ *p* < 0.01, ^###^ *p* < 0.001 compared with LPS/glutamate- and *L. maackianus* MeOH-treated group.

To determine whether the antioxidant effect of *L. maackianus* extract was due to HO-1, the extract was used to treat BV2 microglia and HT22 hippocampus cells for 12 h. Normal HO-1 expression was analyzed by treatment with cobalt protoporphyrin (CoPP), an HO-1 inducer. It was demonstrated that the MeOH extract of *L. maackianus* significantly induced HO-1 expression in BV2 and HT22 cells (Figure 6A,B). Therefore, the effect of this extract on the nuclear translocation of activated Nrf2 was investigated. Nrf2 expression was measured every 0.5 h after treating BV2 and HT22 cells with 100 μg/mL *L. maackianus* extract. It was shown that cytosolic Nrf2 translocated to the nucleus in both BV2 and HT22 cells (Figure 6C,D). Additionally, experiments were performed with the selective HO-1 inhibitor tin protoporphyrin-IX (SnPP) to determine whether the anti-inflammatory effects of *L. maackianus* were related to HO-1 expression. After pretreatment with extract in the presence or absence of 50 μM SnPP, the BV2 and HT22 cells were treated with LPS (1 µg/mL) and glutamate (10 mM) for 24 h. Thus, it was shown that *L. maackianus* extract reduced nitrite production more in LPS-induced BV2 cells treated with the extract alone than when treated with the extract and SnPP together (Figure 6E). In addition, based on the result that the MeOH extract of *L. maackianus* induces HO-1 protein expression, we investigated the neuroprotective properties of antioxidant stress in glutamate-induced HT22 cells. It was determined that the neuroprotective effect was better in glutamate-induced HT22 cells when only the extract was administered than when the extract and SnPP were administered simultaneously (Figure 6F). ROS reduction using the extract was also reversed after SnPP treatment (Figure 6G).

### 3.7. Antioxidant Effect of L. maackianus Extract in Zebrafish Stimulated with H_2_O_2_

**Figure 7:**
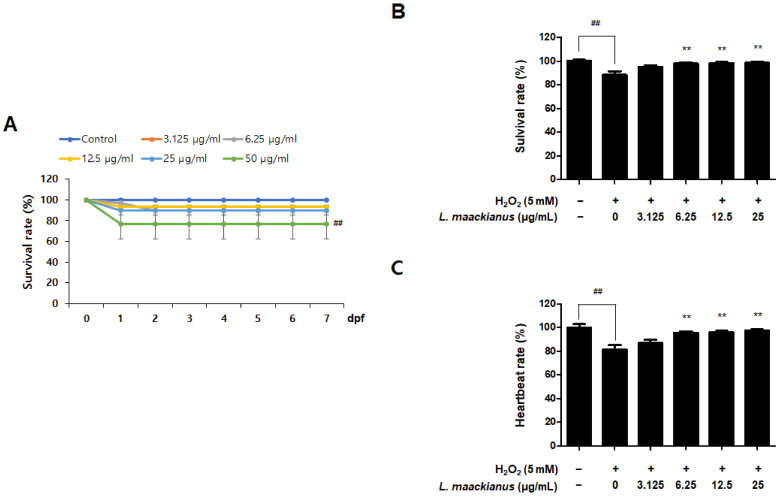
Toxicity evaluation (**A**) and hydrogen peroxide (H_2_O_2_)-induced oxidative toxicity protective effect (**B**) and oxidative stress-induced heart rate reducing effect (**C**) of *L. maackianus* MeOH extract in a zebrafish model. The treatment concentrations used for toxicity evaluation were 3.125–50 μg/mL, and days post fertilization (dpf) was measured daily for 7 days. The results of oxidative stress due to H_2_O_2_ were measured on day 7 after treatment with 5 mM H_2_O_2_ and 3.125–25 μg/mL of MeOH extract. Data are presented as the mean ± standard deviation for three independent experiments. Unprocessed controls were included. ^##^ *p* < 0.01 compared to control. ** *p* < 0.01 compared with H_2_O_2_-treated group.

It has been shown in in vitro experiments that the MeOH extract of *L. maackianus* has antioxidant and anti-inflammatory effects. Therefore, a zebrafish model was treated with H_2_O_2_ and the extract to investigate its potent antioxidant effects in vivo. First, a toxicity assessment was performed to determine the treatment concentration (Figure 7A). The survival rate was significantly reduced at 50 µg/mL. Therefore, the experiments were performed at concentrations of 3.125, 6.25, 12.5, and 25 µg/mL. To measure the antioxidant effect of *L. maackianus* extract, the survival rate was measured on day 7 after treatment with 5 mM H_2_O_2_ and each concentration (3.125–25 µg/mL) of the extract (Figure 7B). It was shown that the survival rate was significantly decreased in the control and H_2_O_2_ treatment groups, and the survival rate was significantly increased in the extract treatment groups. Changes in heart rate due to oxidative stress were also measured (Figure 7C). The H_2_O_2_ treatment group showed a significant decrease in heart rate compared to that of the control group.

**Figure 8:**
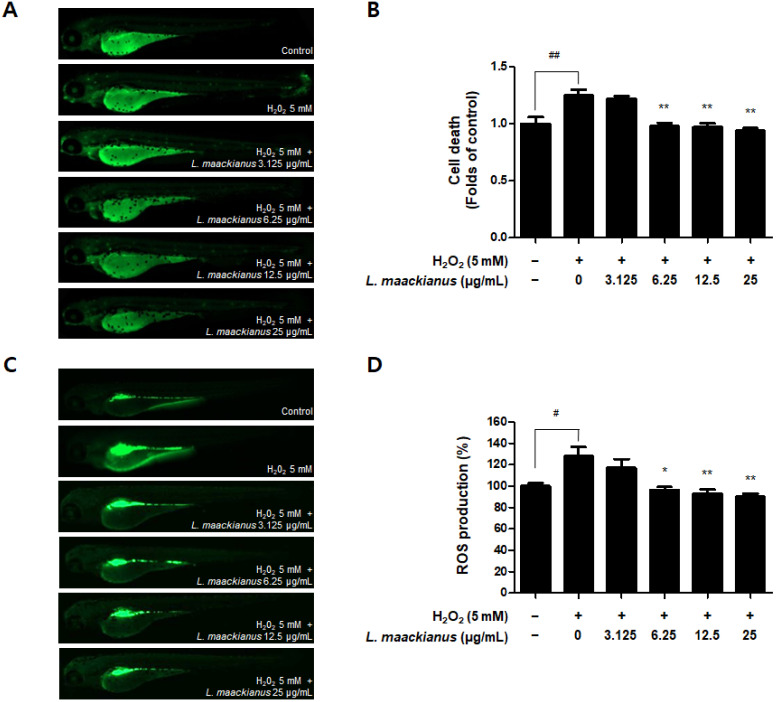
Cytoprotective effect (**A**,**B**) and reactive oxygen species (ROS) generation inhibitory effect (**C**,**D**) of *L. maackianus* MeOH extract in a H_2_O_2_-treated zebrafish model. Results were assessed after 7 d treatment with 5 mM H_2_O_2_ and extract (3.125–25 µg/mL). Data are presented as the mean ± standard deviation for three independent experiments. Unprocessed controls were included. ^#^ *p* < 0.05, ^##^ *p* < 0.01 compared to control. * *p* < 0.05, ** *p* < 0.01 compared to H_2_O_2_-treated group.

Additionally, cell death and ROS production in zebrafish were measured using a fluorescence microscope. Figure 8A,B shows that cell death was significantly increased in the H_2_O_2_ treatment group compared to that in the control group. However, the extract significantly reduced death at 6.25, 12.5, and 25 µg/mL. Figure 8C,D shows the results of evaluating ROS production. Compared to that in the control group, ROS production was significantly increased in the H_2_O_2_ treatment group. However, the extract significantly reduced ROS production at 6.25, 12.5, and 25 µg/mL.

### 3.8. Anti-Inflammatory Effect of L. maackianus Extract in Zebrafish Model Stimulated with LPS

**Figure 9:**
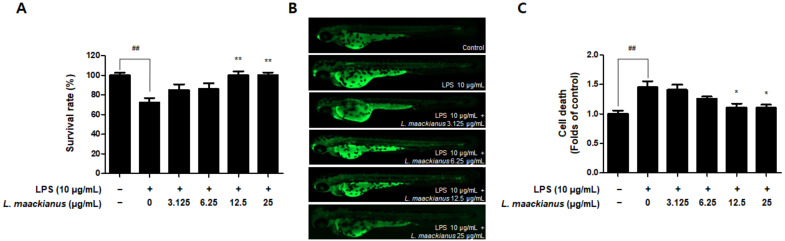
Anti-inflammatory (**A**) and cytoprotective effect (**B**,**C**) of *L. maackianus* (MeOH) extract in LPS-treated zebrafish model. Measurement results after 7 d treatment with 10 µg/mL LPS and extract (3.125–25 µg/mL). Data are presented as the mean ± standard deviation for three independent experiments. Unprocessed control groups were included. ^##^ *p* < 0.01 compared to control. * *p* < 0.05, ** *p* < 0.01 compared to LPS-treated group.

It was shown that the *L. maackianus* MeOH extract had an antioxidant effect in the H_2_O_2_-induced zebrafish model. The zebrafish model has the advantage that it can easily demonstrate the induction of inflammatory responses and oxidative stress. Therefore, LPS-stimulated zebrafish were treated with the extract to investigate its anti-inflammatory effects. The extract was administered at 3.125–25 µg/mL, as determined during the toxicity evaluation (Figure 9A). First, the survival rate was measured on day 7 after LPS treatment (Figure 9A). The survival rate was significantly decreased in the control and LPS-treated groups and significantly increased in the groups treated with 12.5 and 25 µg/mL extract. Cell death was measured using a fluorescence microscope (Figure 9B). Cell death was significantly higher in the LPS-treated group than in the control group. However, it was shown that the extract significantly reduced cell death at 12.5 and 25 µg/mL.

**Figure 10:**
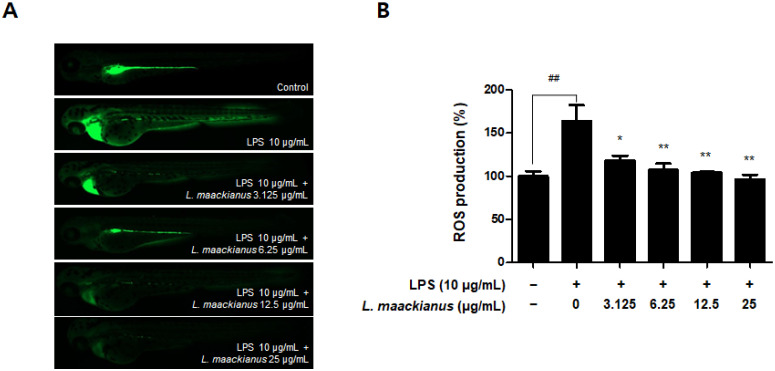

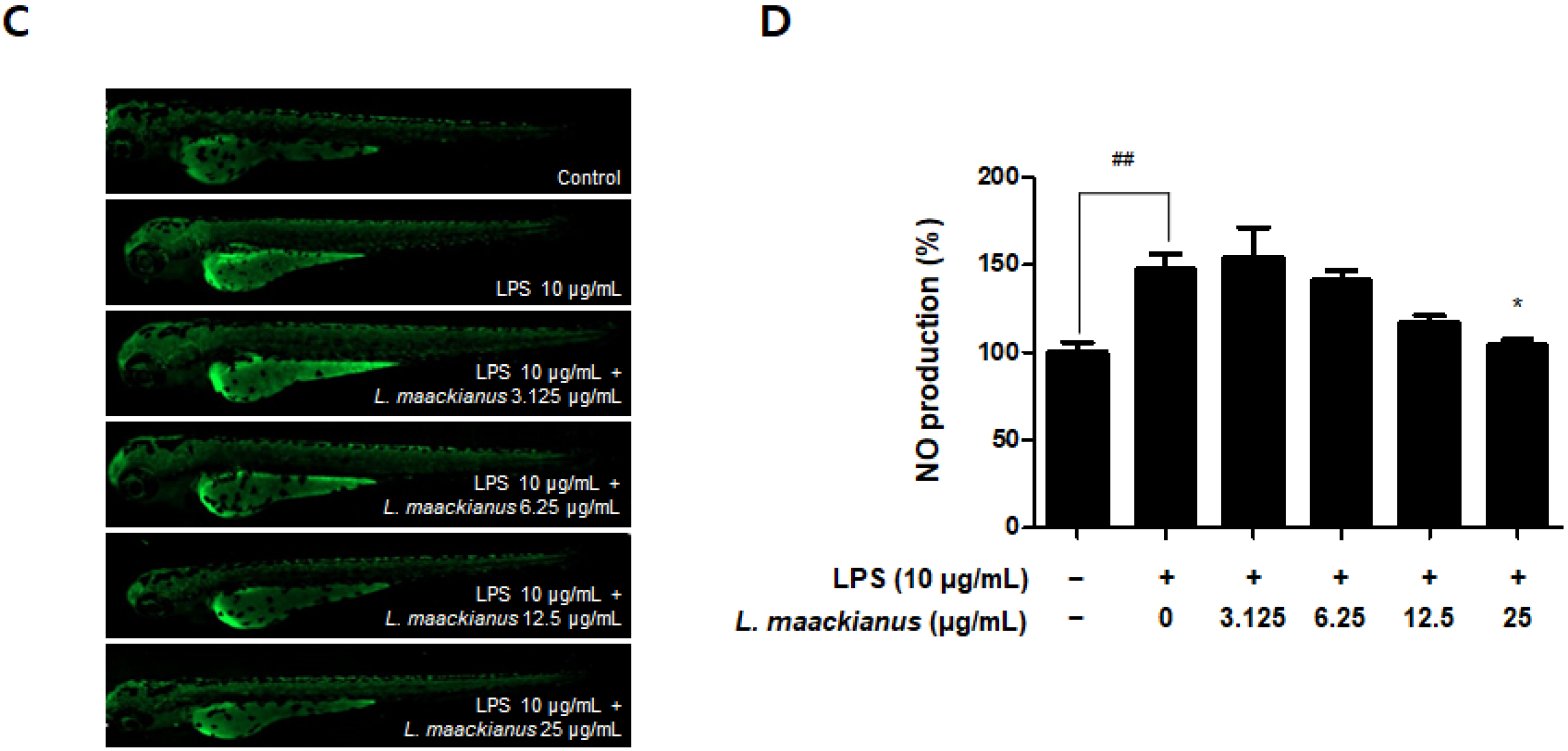
Reactive oxygen species (ROS) (**A**,**B**) and nitric oxide (NO) generation inhibitory effect (**C**,**D**) of *L. maackianus* MeOH extract in lipopolysaccharide (LPS)-treated zebrafish model. Results were obtained after 7 d of treatment with 10 µg/mL LPS and extract (3.125–25 µg/mL). Data are presented as mean ± standard deviation for three independent experiments. Unprocessed controls were included. ^##^ *p* < 0.01 compared to control. * *p* < 0.05, ** *p* < 0.01 compared with LPS-treated group.

As shown in Figure 10A,B, ROS production was significantly increased in the LPS-treated group compared to that in the control group. However, ROS production was significantly reduced in all groups treated with the extract. As shown in Figure 10C,D, NO production was significantly increased in the LPS-treated group compared to that in the control group. However, NO production was reduced only in the group treated with the extract at 12.5 and 25 µg/mL.

## 4. Discussion

*Lycopus maackianus* Makino belongs to the Labiatae family, which includes various medicinal plants. However, there are no studies on the components of *L. maackianus* or reports on its neuroprotection and anti-neuroinflammation effects. In the present study, we profiled the components contained in the MeOH extract of *L. maackianus* using UHPLC–TOF-HRMS and evaluated its neuroprotective action on glutamate-induced HT22 cells and its anti-neuroinflammatory effects on LPS-induced BV2 cells.

First, experiments were conducted on the constituents of *L. maackianus* extract. Many compounds were detected after fingerprinting using UHPLC–TOF-HRMS (Table 1). Most of the detected compounds were unusual compounds that are not commonly seen in Labiatae, and many studies have not been conducted on them. However, some compounds have also been reported to be biologically active. 5,6-Epoxyeicosatrienoic acid(5,6-EET) has been investigated for its specific contribution to transient receptor potential (TRP) channel activation in nociceptive neurons and its consequences for nociceptive processing [25]. In addition, 5,6-EET is known to exhibit vasodilation, anti-inflammatory and anti-platelet aggregation effects as one of the information transmitters produced by epoxygenase in the cytochrome P450 pathway of arachidonic acid [26]. 1-Palmitoyl-sn-glycero-3-phosphocholine may be associated with cognitive ability in patients with mild to moderate Alzheimer’s disease (AD) with *APOE4*^−/−^ [27]. Pheophorbide a is one of the most commonly studied chlorophyll breakdown products owing to its antioxidant and anti-inflammatory activities [28]. Pheophorbide a is used as a photosensitizer and induces significant antitumor effects in several types of tumor cells [29]. According to previous reports, these three compounds may play an important role in the neuroprotective and neuroinflammatory effects of the MeOH extract of *L. maackianus*. In addition, the results on the compounds identified in the UHPLC–TOF-HRMS profiling analysis are meaningful because they are related to the activity of the *L. maackianus* extract.

Next, the antioxidant activity of the MeOH extract of *L. maackianus*, whose activity antioxidant has not been reported to date, was analyzed by performing DPPH and ABTS assays. These methods were used as a screening tool for measuring the antioxidant effect of the extract. The DPPH radical scavenging assay measures the reducing power of a substance using the electron-donating ability of DPPH, which is a relatively stable radical that can become a stable diamagnetic molecule by receiving electrons or hydrogen radicals [30]. ABTS is commonly used as a substrate, and H_2_O_2_ can be used to assess the reaction kinetics of peroxidases. Therefore, the ABTS assay can also be used to indirectly track the kinetics of enzymes involved in the generation of H_2_O_2_ and to quantify the amount of H_2_O_2_ [31]. Thus, the antioxidant effect of *L. maackianus* extract in neuronal cells was determined using the radical scavenging abilities of DPPH and ABTS. *L. maackianus* extract showed increased DPPH and ABTS free radical scavenging activity in a concentration-dependent manner (Figure 1).

Oxidative stress and neuroinflammation are known important factors involved in the pathogenesis of neurodegenerative diseases. NO is a neurotransmitter in the CNS and can cause the release of inflammatory substances and cytokines when overproduced, leading to neuritis and neuronal cell death [32]. NO and PGE_2_ are important mediators involved in the various regulatory functions of the inflammatory process. NO is synthesized from l-arginine and mediates inflammatory responses catalyzed by NOS isoforms [33]. PGE_2_ consists of COX-1, a derivative of arachidonic acid produced by COX-1, inducible COX-2, and inducible COX-2 [34]. Therefore, it was investigated whether *L. maackianus* extract, which was shown to have antioxidant effects, inhibited the production of NO and PGE_2_ (Figure 2B,C). It was shown that the *L. maackianus* MeOH extract had an anti-neuroinflammatory effect. Subsequently, its effect on inhibiting the release of inflammatory cytokines was investigated (Figure 2C,D). The release of IL-6 and TNF-α was suppressed, as expected. Furthermore, we investigated whether *L. maackianus* extract inhibited the expression of the pro-inflammatory proteins iNOS and COX-2 (Figure 3). The expression of iNOS and COX-2 was suppressed by *L. maackianus* extract, leading to the suppression of NO and PGE_2_ production in BV2 cells. This result indicates that the *L. maackianus* extract has an inhibitory effect on neuroinflammation in BV2 microglia.

Furthermore, NF-κB is a pleiotropic transcription factor that regulates the expression of several genes involved in inflammatory responses and stimulates numerous cellular signaling pathways, leading to the increased production of inflammatory cytokines [35,36]. The phosphorylation of IκBα involves kinases such as NF-κB (p65)/p50, which is a crucial step in the activation of the NF-κB pathway, subsequent phosphorylation, and nuclear translocation of p65. The activation of nuclear p65 regulates the transcriptional activation of pro-inflammatory genes such as TNF-α [37,38]. In the present study, *L. maackianus* extract treatment markedly prevented IκBα phosphorylation, reduced nuclear p65 expression, and inhibited NF-κB translocation in LPS-stimulated BV2 cells (Figure 4). The results suggested that there is a possibility that this extract inhibits the production of neuroinflammatory factors by inhibiting the nuclear translocation of NF-κB-p65 in LPS-induced BV2 cells.

Glutamate-induced oxidative stress is a causative factor of neuronal cell death in neuropathological conditions. High concentrations of glutamate trigger oxidative stress, which contributes to neuronal cell death in neurodegenerative diseases [39]. Therefore, a cell protection assay was performed to assess the antioxidant activity of *L. maackianus* extract against oxidative stress in glutamate-induced HT22 hippocampal cells. The results showed that the *L. maackianus* extract significantly ameliorated cell damage in HT22 cells due to glutamate. *N*-acetyl-cysteine (NAC), a strong antioxidant agent, showed an effect similar to that of *L. maackianus* extract (Figure 5B). In neurons, the activation of ionotropic glutamate receptors results in massive calcium entry, calcium overload in mitochondria, energy failure, excessive ROS formation, and, eventually, cell death [40,41]. In the present research, the ROS result further verifies the antioxidant effect of *L. maackianus* extract; the increased ROS production following glutamate in HT22 cells was reversed after *L. maackianus* extract treatment (Figure 5C), which also indicates that the extract has a protective effect in neurons. The intracellular (Ca^2+^) also needs be studied in future research.

Next, the antioxidant, anti-neuroinflammatory, and neuroprotective effects of the *L. maackianus* extract were studied. Heme oxygenase (HO) is a component of the endogenous cell defense against oxidative stress. Two major HO isoforms, HO-1 and constitutive HO-2, the products of separate genes, catalyze heme degradation by similar mechanisms [42,43]. HO-1 is induced by a variety of cell- and species-dependent stress factors, including oxidative stress, and regulated at the level of gene transcription via multiple stress- and antioxidant-response regulatory elements in the promoter region of the HO-1 gene, and the cytoprotective effects of HO-1 have been demonstrated in neurons [44,45]. HO-2 is also necessary for oxidative stress, but HO-1 is more common and well known for the protection of neurons. Nrf2 is the most widely studied Nrf family member. In the case of Nrf2, Nrf2a and Nrf2b have been sub-functionalized, where Nrf2a is a canonical activator of ARE targets and Nrf2b is a negative regulator of several crucial genes, including p53 and HO-1. The activation of the Nrf-2/HO-1 pathway is a critical therapeutic target for cytoprotective strategies [46,47]. Several reports have suggested that the activation of Nrf2/HO-1 is beneficial against neuroinflammation by inhibiting reduced nicotinamide adenine dinucleotide phosphate oxidase, the primary enzyme responsible for microglial ROS release and reactive quinones [48,49]. As expected, the results showed that the *L. maackianus* extract upregulated HO-1 expression in both BV2 and HT22 cells (Figure 6A,B). In addition, the nuclear Nrf2 content increased significantly in the *L. maackianus* extract-treated group, which indicated that the *L. maackianus* extract significantly activated the nuclear translocation of Nrf2 compared to that in the control group in BV2 and HT22 cells (Figure 6C,D). To further evaluate whether this protective effect is mediated by HO-1, both *L. maackianus* extract and SnPP (HO-1 inhibitor) were used to treat LPS-induced BV2 and glutamate-induced HT22 cells. As expected, the *L. maackianus* MeOH extract reduced nitrite production in BV2 cells and protected HT22 cells from oxidative stress, and the HO-1 inhibitor reversed these beneficial effects. This finding suggests that the neuroprotective and neuroinflammatory effects of *L. maackianus* extract are significantly regulated through the Nrf2/ HO-1 signaling pathway. Meanwhile, Nrf2 also protects microglia from cellular stress by activating antioxidative enzymes such as NAD(P)H: quinone oxidoreductase (NQO1). NQO1 functions as quinone reductase by reducing quinones to hydroquinones, hence preventing the one-electron reduction of quinones that results in the production of radical species [50,51], which can also be studied in further research.

Zebrafish (Danio rerio) are widely used as an in vivo model to investigate biological mechanisms owing to the high similarity between humans and zebrafish, such as genomes, rapid embryogenesis, and high reproductive rates [52]. Zebrafish also have key metabolic organs that can be used to investigate metabolism problems in humans [53], and the advantage of being useful in inducing and investigating strong oxidative and inflammatory responses [54]. In addition, grafting of the zebrafish model among the existing in vivo experiments on Labiatae plants has not been extensively performed. Therefore, we selected zebrafish as an in vivo model to further study the effects of *L. maackianus* extracts, which exhibit antioxidant and anti-neuroinflammatory effects in vitro. This is because various reports exist on the evaluation of antioxidant and anti-inflammatory activities in mouse models with Labiatae, and attempts to apply special biological models have been made. An advantage of the zebrafish model is that the heart rate, ventricular action potential, and electrocardiogram are more similar to that of the human heart than those of the mouse model, making the cardiac electrophysiology of zebrafish more clinically relevant [55,56]. The underlying mechanism of the pathogenesis of oxidative stress-induced neurodegenerative diseases is that ROS and RNS accumulate damaging lipids, proteins, and organelles and disrupt mitochondrial membranes, leading to neuronal cell death [3]. Similar to other ROS, H_2_O_2_ causes oxidative damage to DNA, lipids, and proteins, disrupting the natural function of biomolecules through oxidative conformational changes. Zebrafish are an excellent model for in vivo imaging [4]. The effect of ROS-elevated levels is counterbalanced with a variety of antioxidants, which are divided into two categories, namely, enzymatic and non-enzymatic. Superoxide dismutase (SOD), catalase (CAT), glutathione peroxidase (GTPx), and glutathione transferase (GST) are foremost components of enzymatic antioxidants [57]. This study focuses on finding new non-enzymatic antioxidants for reducing ROS production in both in vivo and in vitro conditions. Therefore, we adopted the zebrafish model to demonstrate definitive antioxidant and anti-inflammatory action through in vitro and in vivo investigations. The antioxidant effects of *L. maackianus* extract in H_2_O_2_-induced zebrafish was investigated using various methods. For this investigation, in vivo electrocardiogram and ex vivo voltage-sensitive fluorescent epicardial and transverse optical mapping of zebrafish hearts during H_2_O_2_ exposure were performed. Thus, it was determined that the survival rate and heart rate were restored to normal when treated with 6.25 µg/mL *L. maackianus* extract. In addition, the cytoprotective effect of *L. maackianus* extract and inhibition of ROS production in H_2_O_2_-induced zebrafish was determined (Figure 7 and Figure 8). Furthermore, the anti-inflammatory effect of the *L. maackianus* extract was shown by the induction of zebrafish with LPS. *L. maackianus* extract showed improved viability and cytoprotective effects at 12.5 µg/mL or more and inhibited ROS generation at all concentrations of the extract (Figure 9 and Figure 10). In the future, the mechanism regarding *L. maackianus* extract’s regulation of ROS should be further explored using more methods, like histochemical staining or morphological analysis.

In addition, NO production was inhibited at 25 µg/mL of *L. maackianus* extract. Thus, significant antioxidant and anti-inflammatory effects of the *L. maackianus* extract were demonstrated in a zebrafish model. These results demonstrated that *L. maackianus* extract has potent antioxidant, anti-neuroinflammatory, and neuroprotective effects in both in vitro and in vivo experiments. However, it is necessary to investigate the correlation between *L. maackianus* MeOH extract and compounds analyzed using UHPLC–TOF-HRMS for antioxidant, anti-neuroinflammatory, and neuroprotective effects in further studies.

## 5. Conclusions

Collectively, *L. maackianus* MeOH extract showed significant antioxidant, anti-neuroinflammatory, and neuroprotective effects in both in vivo and in vitro experiments. Furthermore, these effects were significantly related to the regulation of the Nrf2/HO-1 and NF-κB pathways by the *L. maackianus* MeOH extract. Therefore, *L. maackianus* extract is a potential therapeutic agent for neuronal diseases owing to its antioxidant effect.

## Figures and Tables

**Table 1:** Compounds identified in the MeOH extract of *Lycopus maackianus* using ultra-high-performance liquid chromatography–time-of-flight high-resolution mass spectrometry (UHPLC–TOF-HRMS).

Compound Name	M	RT (min)	*m*/*z* Traces (+)	*m*/*z* Traces (−)	MS^2^
5,6-epoxyeicosatrienoic acid	320.2	32.2	321.2		MS2 (+) [321.2]: 185.9, 211.8, 268.0, 286.4
1-naphthalenecarboxaldehyde	304.2	40.8	305.2		MS2 (+) [305.2]: 112.2, 260.8, 291.6
1-palmitoyl-sn-glycero-3-phosphocholine	495.3	42.8	496.3		MS2 (+) [496.3]: 105.0, 284.4, 478.8
Pheophorbide A	592.3	48.6	593.3		MS2 (+) [593.3]: 434.6, 463.4, 507.1, 534.1
*N*-acetylsulfamerazine	306.1	16.6		305.1	MS2 (−) [305.1]: 60.0, 97.2, 225.4, 305.5
(−)-12-hydroxyjasmonic acid	226.1	19.5	227.1		MS2 (+) [227.1]: 132.5, 149.3, 191.3, 210.1
Harderoporphyrin	608.3	48.1	609.3		MS2 (+) [609.3]: 531.5, 591.2, 610.2
all-trans-13,14-dihydroretinol	288.2	46.9	289.2		MS2 (+) [289.2]: 95.6, 110.0, 272.3, 289.7
17-methylene-4-androsten-3-one	284.2	36.5	285.2		MS2 (+) [285.2]: 96.0, 105.6, 119.0, 160.0, 211.9, 267.7
Sterebin	338.2	32.0		337.2	MS2 (−) [337.2]: 45.0, 253.8, 271.8, 337.7, 383.8

## Data Availability

The data presented in this study are available within this article.
